# A novel method for extraction, quantification, and identification of microplastics in CreamType of cosmetic products

**DOI:** 10.1038/s41598-021-97557-0

**Published:** 2021-09-10

**Authors:** Soohyun Lee, Tai Gyu Lee

**Affiliations:** Department of Chemical and Biomolecular Engineering, Yonsei University, 50 Yonsei-ro, Seodaemun-gu, Seoul, 03722 Korea

**Keywords:** Environmental chemistry, Environmental impact

## Abstract

The objective of this study was to develop an accessible and accurate analysis method for microplastics that have been unintentionally added to cream cosmetic products. An experiment was performed on three cleansing creams in rich and viscous formulations. A spiked sample was prepared by adding polyethylene (PE) microspheres to the cleansing creams. After removing cosmetic ingredients from the creams using chemical digestion, damage to the PE microspheres was identified using Fourier transform infrared (FT-IR) spectroscopy. Field emission scanning electron microscopy (FE-SEM) images were obtained before and after digestion and used to characterize the morphology of the PE microspheres. The highest digestion efficiency was obtained using a chemical digestion method consisting of heating and stirring a sample in a 10 wt% KOH solution at 55 °C and 300 rpm for 5 days and did not damage the PE microspheres. The Nile red (9-diethylamino-5H-benzo[α]phenoxazine-5-one) staining method was effective in identifying small microplastics (< 106 μm). The optimal staining conditions are 5 μg/ml Nile red in *n*-hexane for green wavelengths.

## Introduction

Microplastics are pieces of synthetic polymer compounds with sizes of 5 mm or lower that; can be intentionally or unintentionally produced^1^ and exposed to the environment through various routes. Recently, countries with advanced cosmetics technologies, such as the United States, the United Kingdom, France, and South Korea, have legally prohibited the intentional use of microplastics in cosmetics^2–8^. Several studies have been performed on the detection of microplastics in cosmetics but were conducted before the ban on the intentional use of microplastics in cosmetics and applied an analysis method developed for intentionally used microplastics^9–12^. These studies were carried out using cosmetic products containing microbeads, and the pretreatment method was heating and stirring the products with only purified water until complete dissolution. After pretreatment was completed, the color, shape, polymer composition, size, density, and weight of microplastics were analyzed. Currently, most microplastics research is focused on detection in the ocean, sand, and living organisms, and pretreatment using strong acids is mostly performed for the purpose of digesting organic matters^13–20^.

Microplastics, as defined by the Ministry of Food and Drug Safety (MFDS) of South Korea, which has the 9th largest cosmetics market in the world^21^, refer to “solid plastics of 5 mm or less that are ‘intentionally’ added into products for the purpose of exfoliating and cleansing^7^.” Following the MFDS guidelines for the analysis of prohibited ingredients in cosmetics, the following test method was used: 3 g of a product were mixed with 100 ml of purified water and 100 ml of ethanol, and the mixture was homogenized by stirring for at least 10 min. Then, the mixture was passed through a metal filter, and the filter and retained particles were oven-dried at 50 °C. The retained particles were identified using an FT-IR microscope. Microplastics were identified by comparing the spectrum of the sample particles with the spectra of various plastics recommended by the MFDS.

The MFDS test method has limitations: (1) the cosmetic ingredients in all formulations are not completely removed by pretreatment with only purified water and ethanol; (2) semi-solid substances, such as sucrose or wax in cosmetics, can be misidentified as solids; (3) as was recently reported for bottled water^22–25^, the method is limited to analyzing microplastics that may have been unintentionally added to cosmetics; and (4) the expensive FT-IR microscope and metal filters required for analysis require specialized fabrication, preventing universal applications.

The objective of this study is to find an optimal digestion solution for the pretreatment of cosmetics in cream formulations and develop an analysis method for cosmetics that can detect all intentionally and unintentionally added microplastics with good accessibility and accuracy using the fluorescence of the plastic coloring reagent Nile red (NR).

## Results

### Determination of the optimal chemical digestion solution

The cosmetic products used in this study were cleansing creams in a viscous formulation. Polyethylene (PE) microspheres were used as the target materials and spiked into the cleansing creams on which experiments were conducted. PE is a plastic that has been widely used as a cosmetic ingredient for exfoliating purposes, as well as a material for cosmetic containers.

Experiments were conducted on different solutions by using cleansing cream B, which was the richest formulation. The solutions used in this experiment were HNO_3_ (60%), Acid mix (HNO_3_:HClO_4_ = 4:1, v:v), H_2_O_2_ (35%), KOH (10%), and D.I water. Among those, acid mix is a digestion solution recommended by the Oslo-Paris Commision (OSPAR) where the mechanism by which 15 Governments & the EU cooperate to protect the marine environment of the North-East Atlantic^26^.

Vacuum filtration was performed after chemical digestion, and the filter was completely dried. The PE microsphere particles retained on the filter were counted and stored in a glass vial. Subsequently, the weight of the filter from which the particles were removed was measured. After filtration, the digestion efficiency (%) was calculated using Eq. (1)^27^:

1 $$Digestion\,efficiency \left(\text{\%}\right)=\frac{{W}_{i}-\left({W}_{a}-{W}_{b}\right)}{{W}_{i}}\times 100$$

where W_i_ is the initial weight of the sample (g), W_a_ is the weight of the dry filter after filtration (g) and W_b_ is the weight of the dry filter before filtration (g).

The filtered PE microsphere particles were counted, and the recovery rate (%) was calculated using Eq. (2):

2 $$Recovery\,rates \left(\text{\%}\right)=\frac{{N}_{b}}{{N}_{a}}\times 100$$

where N_a_ is the number of microplastics (MPs) added to the sample and N_b_ is the number of MPs retained on the dry filter after filtration.

**Figure 1:**
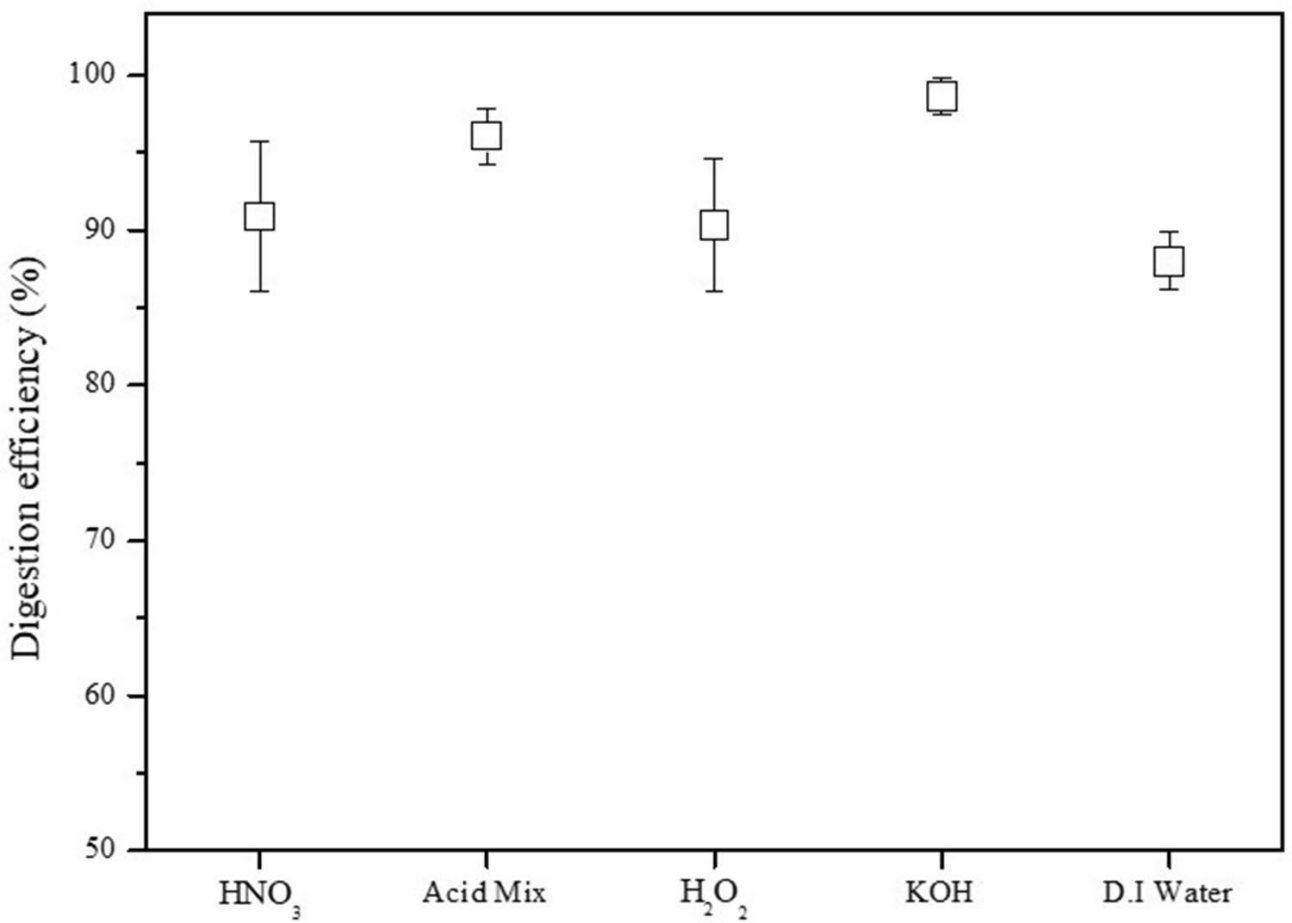
Digestion efficiency using different solutions.

**Figure 2:**
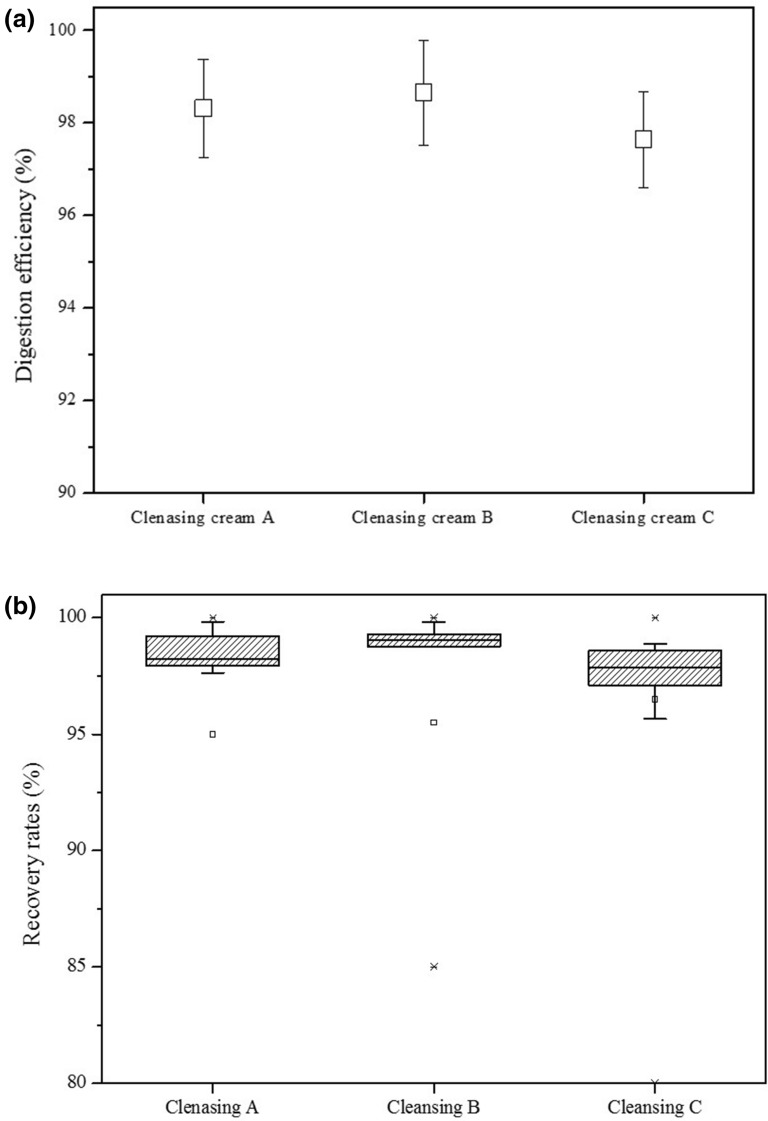
(**a**) Digestion efficiency and (**b**) recovery rates for KOH digestion.

The following digestion efficiencies were determined using the different solutions (Fig. 1): HNO_3_; 90.87 ± 4.81%, Acid Mix; 96.09 ± 1.79%, H_2_O_2_; 90.34 ± 4.26%, KOH; 98.65 ± 1.14%, and D.I water; 88.01 ± 1.87%. D.I water produced the lowest digestion efficiency among the solutions of 90% or lower (Fig. 2a).

A digestion efficiency > 95% is considered to be significant, which was met using the acid mix and KOH. As the acid mix is a mixture of HNO_3_ and HClO_4_, KOH was determined to be the optimal digestion solution considering of cost and convenience of use.

In the filter images obtained after filtration (Supplementary Fig. S2), filtrates with a lumpy cream formulation were observed in solutions other than KOH. All the solutions used in this study can be effectively used to detect microplastics in ocean environmental samples^10,28–33^. In the successful chemical digestion of cosmetic products, the cosmetic ingredients are removed, and only the PE spiked into the product remains on the filter paper after filtration. However, lumpy cream formulations were observed in all the samples digested with acidic solutions. This result suggests that the conventional method of using acidic solutions to detect microplastics in the ocean is not suitable for cosmetic products.

After being confirmed as the optimal digestion solution, KOH was used to digest cleansing creams A–C. A high digestion efficiency > 95% was obtained. Monitoring the loss of 20 PEs added to the creams during filtration showed a recovery rate > 95% (Fig. 2b).

The experiments to determine the digestion solution were performed 5 times per solution, and the experiment using KOH was performed 10 times per cream, for a total of 30 experiments were carried out (Table 1).

**Table 1:** Digestion efficiency (%) of each solutions.

No	HNO_3_	Acid mix	H_2_O_2_	D.I Water	KOH		
	Cleansing cream B	Cleansing cream B	Cleansing cream B	Cleansing cream B	Cleansing cream A	Cleansing cream B	Cleansing cream C
1	89.5650	97.2215	90.3259	87.0357	99.2149	98.6602	98.0961
2	86.0534	96.3140	96.4376	85.4819	99.1104	99.1661	98.8459
3	87.6995	93.8964	91.8881	89.0197	99.2309	96.7133	98.5196
4	98.1920	94.7222	85.1162	88.1208	99.7937	99.0171	98.8298
5	92.8417	98.2797	87.9295	90.3842	98.1719	99.1775	96.4808
6	–	–	–	–	97.6196	99.0990	97.8894
7	–	–	–	–	97.9579	96.2249	97.1106
8	–	–	–	–	97.9772	98.8900	95.3007
9	–	–	–	–	98.2663	99.7936	97.4481
10	–	–	–	–	95.8631	99.7133	97.8300

### Chemical damage test

Chemical weathering of microplastics occurs under environmental conditions, such as in oceans and sand because of UV exposure and abrasion, but does not occur in cosmetics. Thus, chemical damage occurs under inappropriate pretreatment.

**Figure 3:**
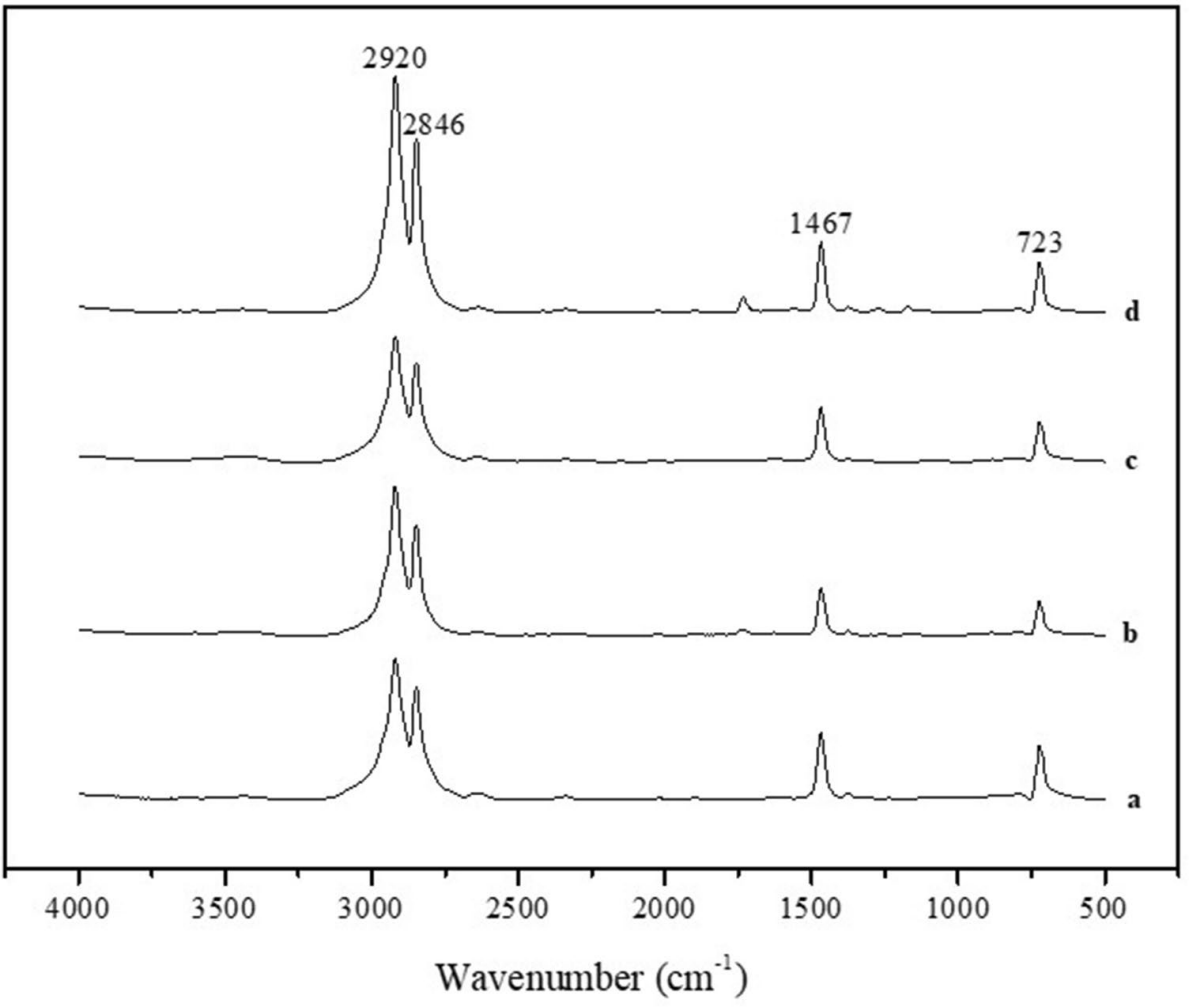
FT-IR spectra of PE microspheres (**a**) before digestion and after KOH digestion in (**b**) cleansing cream A, (**c**) cleansing cream B and (**d**) cleansing cream C.

Damage to the PE mocrospheres was determined by comparing the spectra before chemical digestion and drying and storage following KOH digestion. The measurement results confirmed that the peaks of the PE microspheres after and before pretreatment and the reference peak of the PE microspheres presented by MFDS were consistent (Fig. 3 and Supplementary Fig. S3). This result suggests that no ingredients in the cosmetic formulation remained on the surface because KOH pretreatment was effectively performed without destroying the PE chemical structure.

### Physical damage test

FE-SEM was used to compare the morphology and surface characteristics of PE microspheres before and after pretreatment. First, it was observed whether the overall spherical shape was maintained at low magnification, and the surface properties were then identified at high magnification.

**Figure 4:**
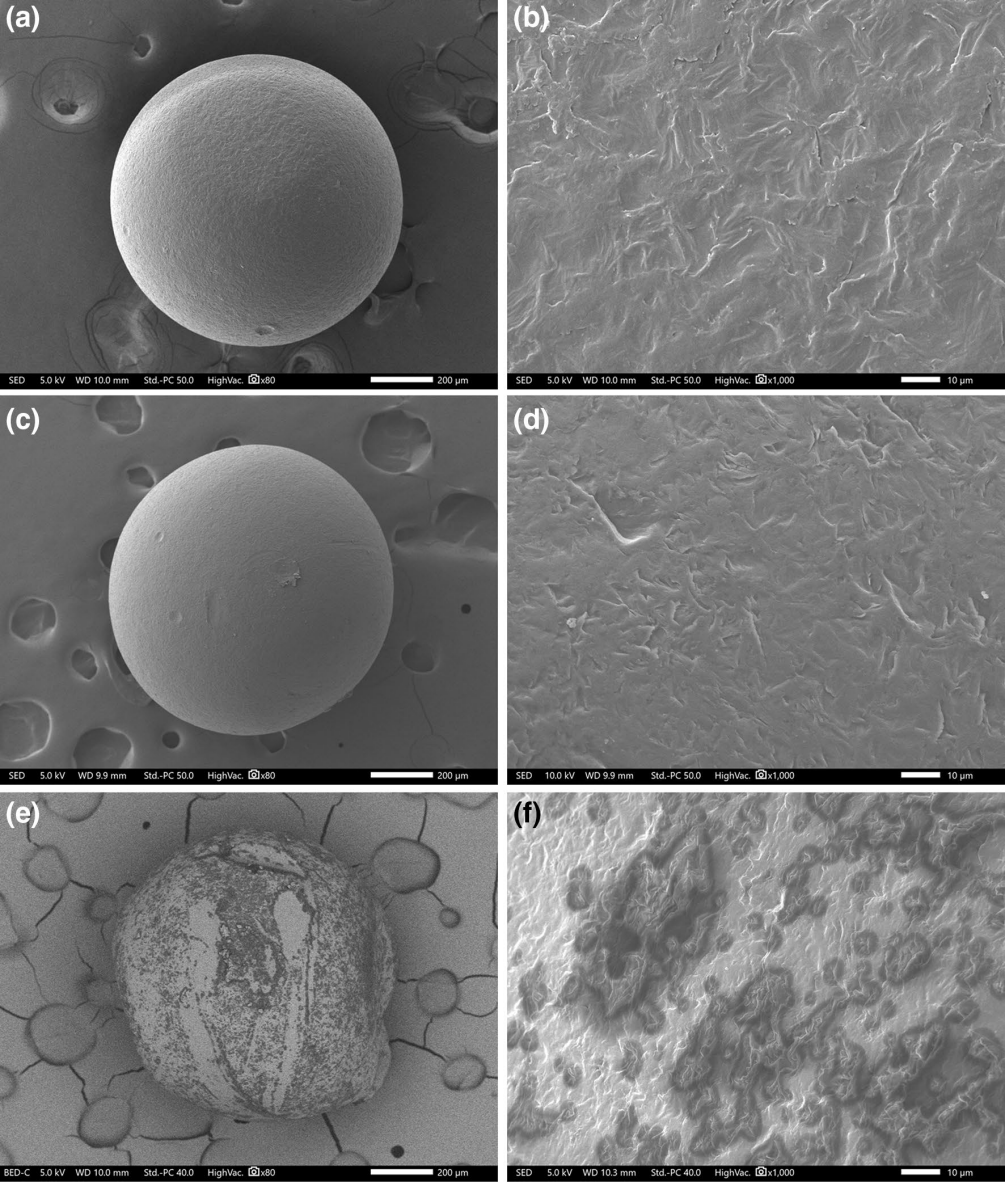
FE-SEM images of PE microspheres before digestion at (**a**) low and (**b**) high magnification; after KOH digestion at (**c**) low and (**d**) high magnification; and after Acid Mix digestion at (**e**) low and (**f**) at high magnification.

As the pretreatment on PE microspheres using KOH solution was effective, no cosmetic formulation ingredients remained on the surface; thus, the images of the PE microspheres obtained after digesting were similar to those obtained before chemical digestion (Fig. 4a–d). By contrast, the ingredients were not properly removed from samples using an acid solution, resulting in irregular and uneven surfaces, and the overall shape had a cut shape, rather than a spherical one (Fig. 4e,f). These results suggest that physical weathering occurred and the acid solution is not suitable for the pretreatment of cosmetic formulations.

### Solvent test for fluorescence analysis

A solvent test is the most basic step in fluorescence analysis using NR. As NR produces different colors depending on the solvent, a solvent should be selected that does not damage the black PC filter used for fluorescence analysis. Three solvents were considered: methanol, *n*-hexane, and chloroform.

Solvent tests were performed using 5 μg/ml working solutions of NR in each solvent. The black PC filter was significantly damaged by chloroform and bleached by methanol (Supplementary Fig. S7).

**Figure 5:**
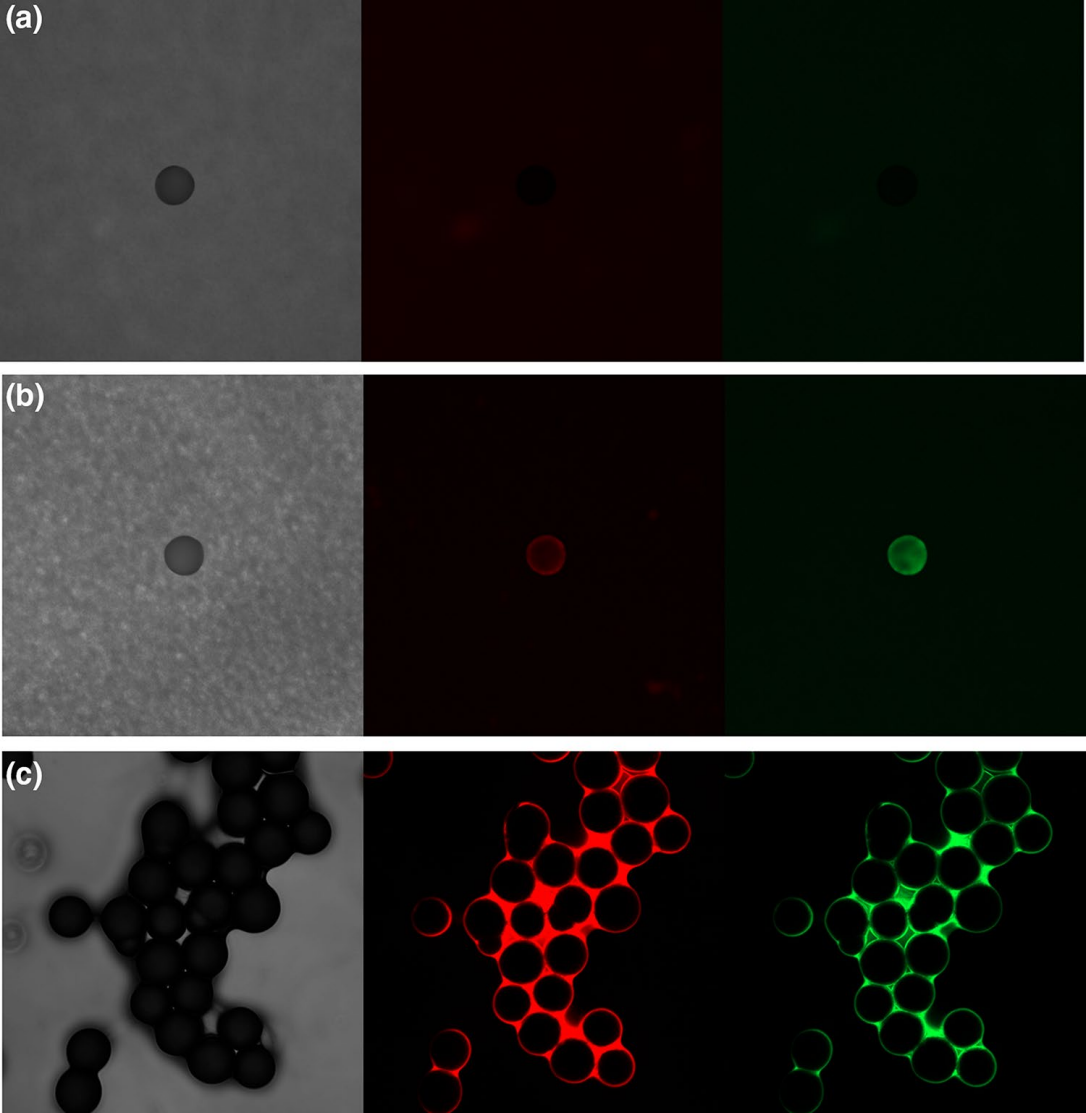
Fluorescent images with shadow (left) and at red wavelengths (middle) and green wavelengths (right). PE microspheres stained with a 5 μg/ml NR working solution in (**a**) methanol, (**b**) *n*-hexane and (**c**) chloroform.

A dyed black PC filter was placed on a glass slide, and a small quantity of PE microspheres was placed on the filter and covered with a cover glass. The edge of the cover glass was fixed with grease to prepare the sample for fluorescence analysis (Fig. 5). The analysis using chloroform failed because the black PC filter melted, such that failed to act as a background, and methanol was not visualized by fluorescence. Only *n*-hexane was clearly observed. Thus, *n*-hexane is the least decomposed of the investigated solvents and was selected for fluorescence analysis.

### Determination of excitation and emission wavelengths

To observe the dyed PE microspheres, experiments were conducted at three wavelengths commonly used in fluorescence analysis^34^. First, a shadow image was obtained to identify the shape and location of the PE microspheres and used to determine whether the PE microspheres could be identified from fluorescence at different wavelengths (Supplementary Fig. S8). The PE microspheres could be visualized at both red and green wavelengths but not at blue wavelengths. Thus, experiments were conducted using different concentrations of PE microspheres determine the optimal conditions at red and green wavelengths.

### Optimization of staining concentrations

**Figure 6:**
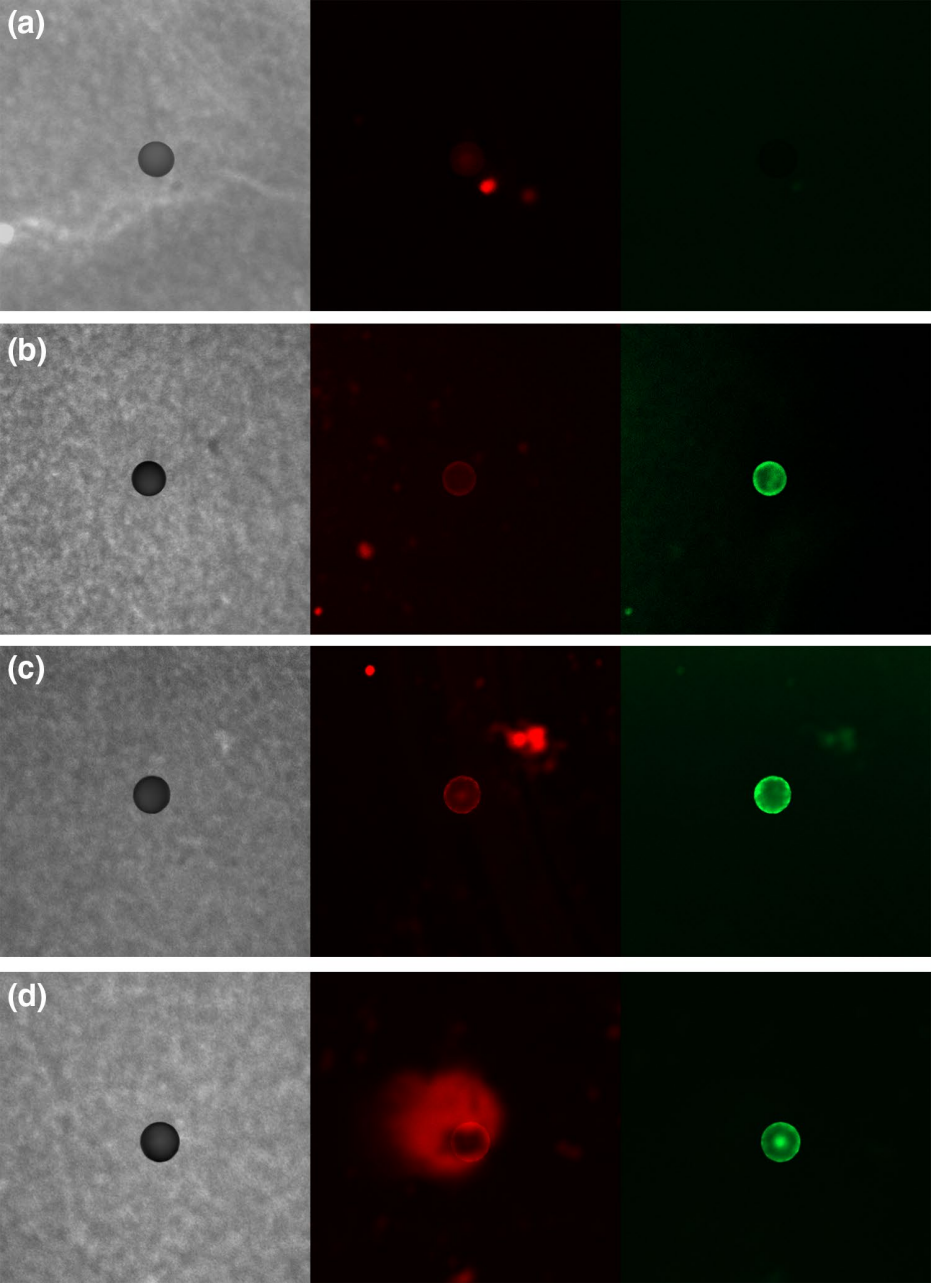
Fluorescent images of PE microspheres stained using NR concentrations of (**a**) 1 μg/ml, (**b**) 5 μg/ml, (**c**) 10 μg/ml and (**d**) 20 μg/ml.

A stock solution of 1000 μg/ml NR was prepared and diluted with *n*-hexane to prepare 1, 5, 10, and 20 μg/ml solutions. Figure 6 shows the observed images for each concentration. Very weak fluorescence was emitted by the 1 μg/ml solution, making it difficult to visualize the PE microspheres. For green wavelengths, PE microspheres were clearly observed at NR concentration of 5 μg/ml or higher whereas for red wavelengths, PE microspheres could be observed at 10 μg/ml or higher. For green wavelengths, there was no significant difference in visualizing PE microspheres at 5 μg/ml or higher, whereas fluorescence appeared in the background and a blurring phenomenon was observed with increasing concentration for red wavelengths. Thus, the optimal NR staining condition for observing the microspheres was determined to be 5 μg/ml NR for green wavelengths, considering cost efficiency and the fluorescence intensity.

PE microspheres dyed under optimal conditions can be clearly identified and counted using a tile mode that can capture the entire filter shape (Supplementary Fig. S9).

## Discussion

The objective of this study was to develop an accessible and accurate method to detect unintentionally added microplastics in cream formulations. Cosmetic products come in various formulations ranging from toners to creams. In dilute formulations with a high moisture content, chemical digestion can be successfully performed using only purified water, whereas rich formulations have the disadvantage of a high oil content, resulting in the complete removal of all ingredients. Although it would be interesting to apply the proposed method to cosmetic products with real microbeads, but the sale and manufacturing of cosmetics containing plastic microbeads is currently prohibited by law and/or reguations, such that samples cannot be obtained. Therefore, experiments were performed using cleansing creams in rich and viscous formulations and PE which was widely used in the form of microbeads in cosmetics.

In this study, PE microspheres were added to viscous cleansing creams, and the resulting mixtures were chemically digested with diggerent solutions to pretreat organic matter to determine the optimal solution. The highest digestion efficiency was obtained by heating and stirring the sample with a 10% KOH solution at 55 °C and 300 rpm for 5 days. The results of FT-IR and FE-SEM analyses confirmed that chemical digestion did not cause physical or chemical damage to the added PE microspheres. When chemical digestion was performed with acidic solutions used to detect microplastics in the ocean^35–39^, ineffective pretreatment resulted in cosmetics ingredients remaining on the surfaces of the PE microspheres. This result suggests that the conventional method using acidic solutions to detect microplastics in the ocean is not suitable for cosmetic products.

The NR staining method selectively stains microplastics^40–43^ that can then be visually analyzed using microscopy; thus, this method facilitates more accessible and accurate analysis than the conventional method without requiring a specialized instrument, such as an FT-IR microscope. Among the investigated solvents, fluorescence was clearly observed without damaging the black PC filter in *n*-hexane. As *n*-hexane has a low solubility for NR powder, a 1000 μg/ml standard stock solution of NR was prepared with acetone^34^, and a working solution was prepared by dilution with *n*-hexane for use in the experiments. The dyed PE microspheres were most clearly identified at green wavelengths, and the optimal staining concentration was 5 μg/ml NR. Fluorescence analysis using NR was particularly effective in identifying small microplastics (< 106 μm) and could therefore improve the accuracy of the analysis by identifying microplastics that could be missed or lost by filtration because of their small size.

As only PE was used in this study, further study is necessary using other polymers, such as polypropylene (PP) and methyl methacrylate (PMMA). Using purified water to rinse the filter paper used to perform an initial filtration after chemical digestion using KOH solution, followed by performing a second filtration using a black PC filter would be reduce microplastics loss and increase the accuracy of detection, identification, and counting using the proposed analysis method.

## Methods

### Materials

The target microplastics used in this study were clear polyethylene microspheres (Cospheric LLC, Santa Barbara, CA, USA). Hydrogen peroxide (35%, DAEJUNG, Siheung-si, South Korea), nitric acid (60%, DAEJUNG, Siheung-si, South Korea), perchloric acid (70%, DAEJUNG, Siheung-si, South Korea), sodium hypochlorite (9%, DAEJUNG, Siheung-si, South Korea), and potassium hydroxide (84%, DAEJUNG, Siheung-si, South Korea) were used as pretreatment solvents.Filtration was performed using 90-mm diameter Whatman Grade 40, Ashless filter paper, and 25-mm diameter black polycarbonate (PC) filter paper (0.8 μm) was used for fluorescence analysis. The dyeing reagents used in the fluorescence analysis were Nile red (Sigma-Aldrich, Saint Louis, USA), acetone (99.5%, DAEJUNG, Siheung-si, South Korea), *n*-hexane (95%, DAEJUNG, Siheung- si, South Korea), methyl alcohol (99.8%, JT Baker, Phillipsburg, NJ, USA), and chloroform (99.8%, DAEJUNG, Siheung-si, South Korea).

### Sample preparation

Three viscous creams from the cleansing product group were used in the experiments. As this study is not affiliated with a specific brand name, the products were labeled Cleansing Creams A-C to avoid any legal issues. Cleansing Creams A-C were selected from the three most sold products in skin care-cleansing-cleansing cream/milk in domestic drug stores.

### Chemical digestion

To determine the solution for use in chemical digestion, 2 g of a cleansing cream was placed into a 250 ml beaker, to which 20 PE microspheres (850–1000 μm) were added. Then, 50 ml of each digestion solution was added to the beaker, and the mixture was stirred at 50–55 °C for 5 days at 300 rpm. The beaker was sealed to prevent evaporation and contamination.

### Filtration

After chemical digestion was completed, the sample was cooled approximately 30 °C and filtered under reduced pressure. The filter was then transferred to a glass petri dish (90 mm in diameter) using tweezers, covered with a lid, and dried at 40 °C to completely remove moisture. Using a plastic petri dish generates static electricity that can result in the loss of the filtered PE microsphere particles. To prevent this loss, a glass petri dish should be used instead of a plastic dish. The PE microsphere particles on the completely dried filter were counted and stored in a glass vial. To calculate the digestion efficiency, the weights of the filter and petri dish were measured. This step enabled the percentage of undigested cleansing cream remaining in the filter to be calculated, indicating whether digestion was successful.

### Spectroscopy

After chemical digestion, FT-IR spectroscopy was used to check for damage to the PE microspheres. The spectra of the PE microspheres before and after digestion and the reference PE spectra provided by the MFDS were compared. The FT-IR spectra were obtained in transmittance mode by using a Vertex 70 FT-IR spectrometer. The FT-IR spectra were analyzed from 500 to 4000 cm^−1^, and 16 scans were performed to obtain each spectrum.

### Field emission scanning electron microscopy

To identify the shape and surface characteristics of the PE microspheres before and after pretreatment, images were obtained by using FE-SEM. An FE-SEM IT-500HR was used, and the operating voltage was 5.0 kV. Before observation, the samples were coated with Pt for 240 s by using a sputter coater. The samples compared under the same conditions, that is, the overall spherical shape was observed at × 80 magnification, and the surface characteristics were observed at × 1000 magnification.

### Fluorescence analysis using Nile red staining

Laser scanning confocal microscopy was performed using a Zeiss LSM 980 for fluorescence analysis. The operation laser power was 2–5%, the pinhole size was 100 μm, the master gain value was 550–600 V, and a digital offset was not used.

### Solvent test for fluorescence analysis

The solvent used in the experiments should not damage the black PC filter used for fluorescence analysis. Three solvents were considered: methanol, *n*-hexane, and chloroform. A few drops of each solvent were dropped onto the filter, and the color change and the degree of damage to the filter were observed.

### Excitation and emission wavelengths

There is a small difference in the excitation/emission wavelengths of NR depending on the polarity of the solvent and dyeing conditions. To observe dyed organic matter, experiments were conducted with three types of wavelengths commonly used in fluorescence microscopy: red: excitation wavelength (ex.) 565 nm and emission wavelength (em.) 753 nm; green: (ex.) 460 nm and (em.) 568 nm; and blue: (ex.) 410 nm and (em.) 597 nm.

### Staining of microplastics using various concentrations of Nile red solution

NR powder was dissolved in each solvent to prepare stock solutions of 1000 μg/ml that were further diluted with solvent to prepare working solutions of various concentrations. All the prepared solutions were syringe filtered before use. A small quantity of PE microspheres (90–106 μm) were placed on a black PC filter, the filter was dyed with 400 μl of a working solution, air-dried for 5 min, and a preparat was fabricated and analyzed using a Zeiss LSM 980. Observation was performed immediately after production to prevent reduction in the NR fluorescence.

## Supplementary Information

Supplementary Information.

## Data Availability

The data that support the findings of this study are available in the paper and its Supplementary Information or from the corresponding author upon request.
