# Supplementary material for: A novel method for extraction, quantification, and identification of microplastics in CreamType of cosmetic products
Source: Sci Rep. 2021 Sep 10;11:18074. doi: 10.1038/s41598-021-97557-0 (PMC8433340; doi:10.1038/s41598-021-97557-0)
Supplement: Supplementary file 1 — Supplementary Information. [file 41598_2021_97557_MOESM1_ESM.docx]

## Supplementary information

# A Novel Method for Extraction, Quantification, and Identification of Microplastics in Cream Type of Cosmetic Products

*Soohyun Lee^1^; Tai Gyu Lee^1*^*

*^1^* *Department of Chemical and Biomolecular Engineering, Yonsei University,*

*50 Yonsei-ro, Seodaemun-gu, Seoul 03722, Korea*

*corresponding author: Department of Chemical and Biomolecular Engineering, Yonsei University; teddy.lee@yonsei.ac.kr


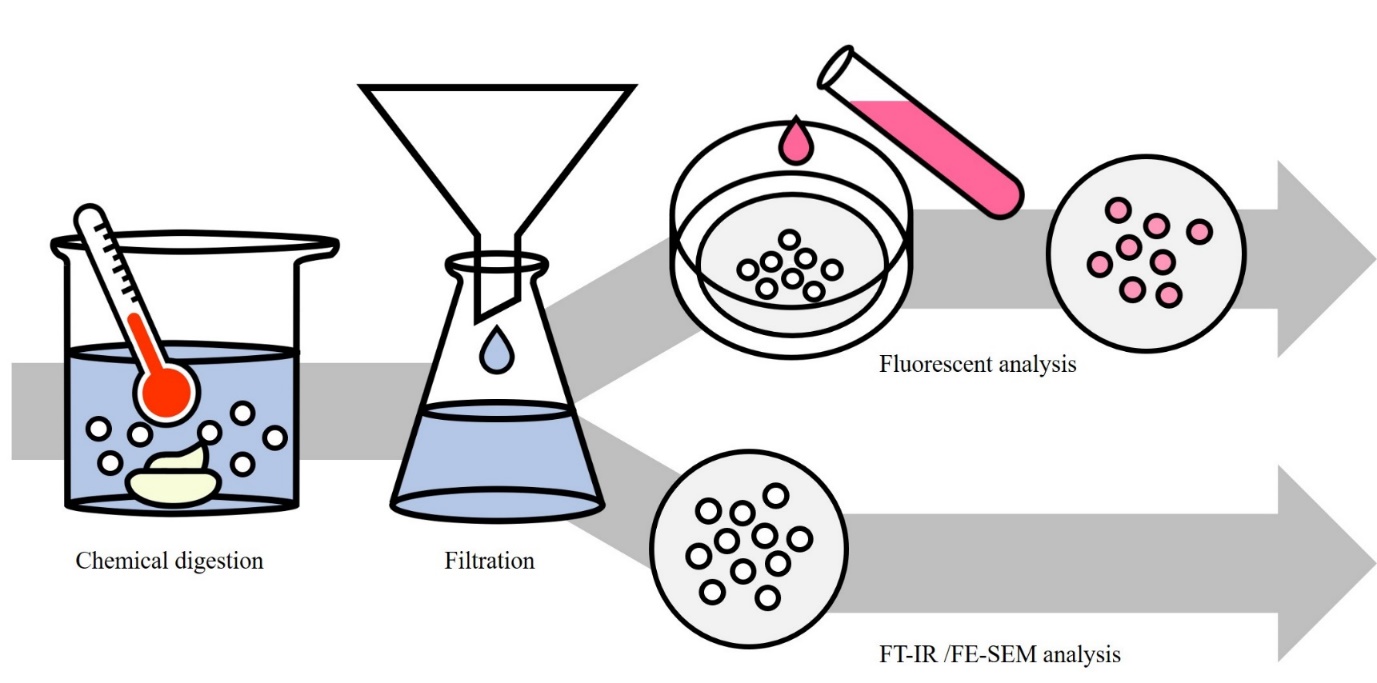


**Figure S1.** Schematic diagram of the analysis process.


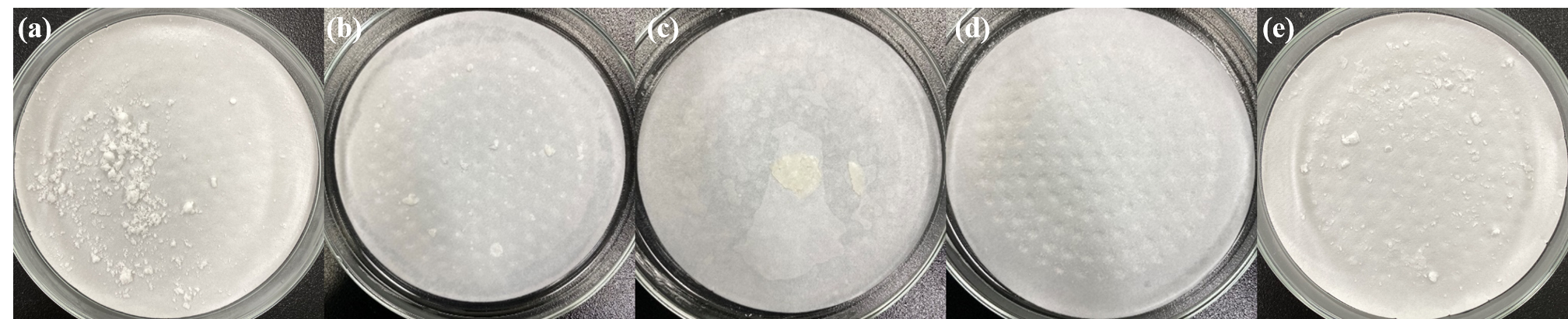


**Figure S2.** Filter images immediately after filtration for each solution. (a) HNO_3_, (b) Acid mix, (c) H_2_O_2_, (d) KOH, (e) D.I water.


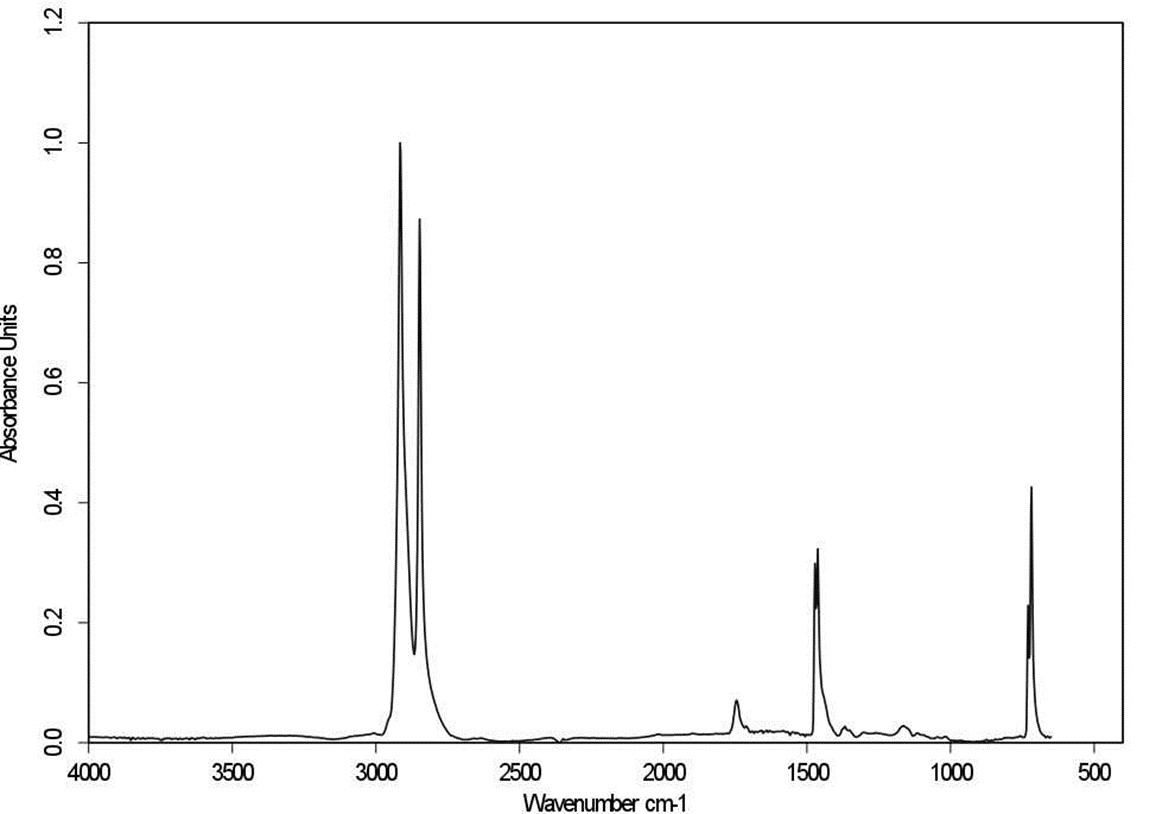


**Figure S3.** FT-IR spectra of polyethylene recommended by the Ministry of Food and Drug Safety of Korea.


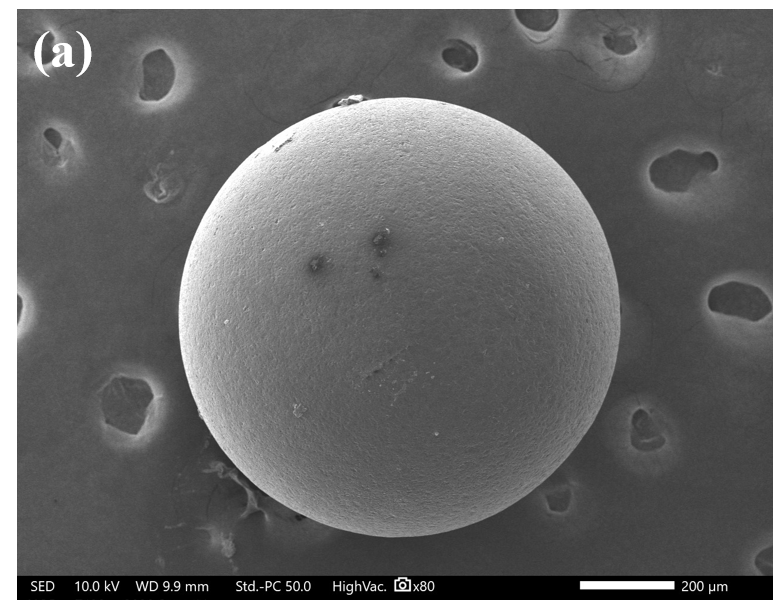


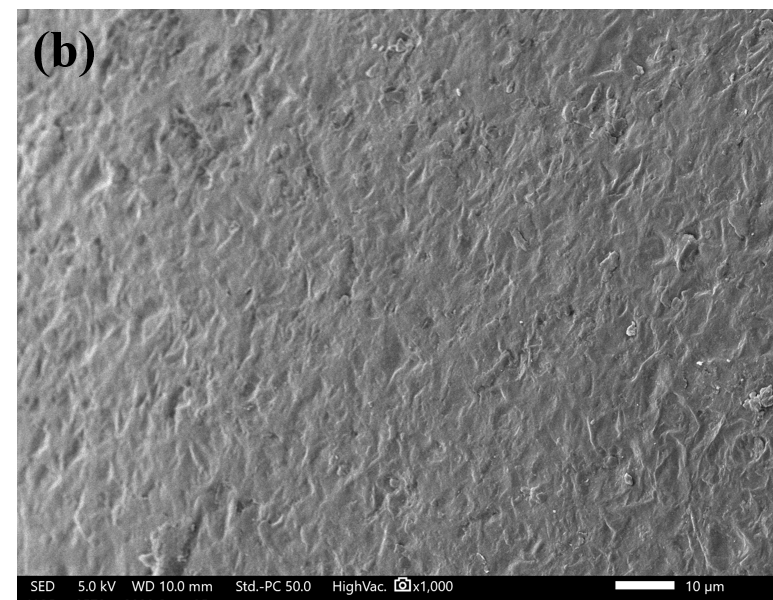


**Figure S4.** FE-SEM images of PE microspheres in cleansing cream B after KOH digestion. (a) At low magnification. (b) At high magnification.


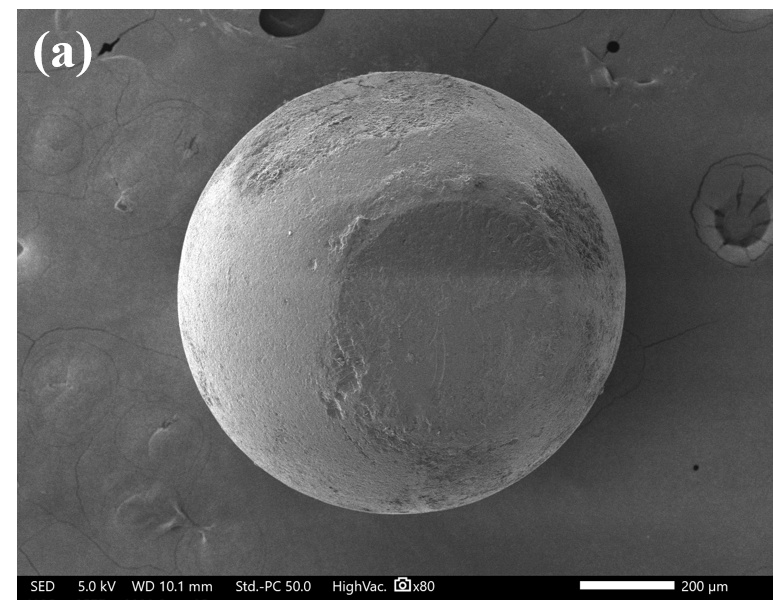


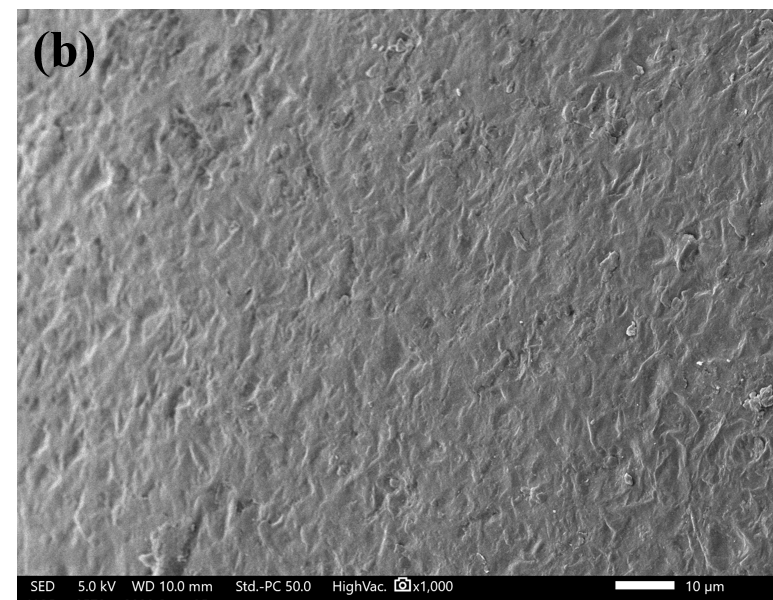


**Figure S5.** FE-SEM images of PE microspheres in cleansing cream C after KOH digestion. (a) At low magnification. (b) At high magnification.


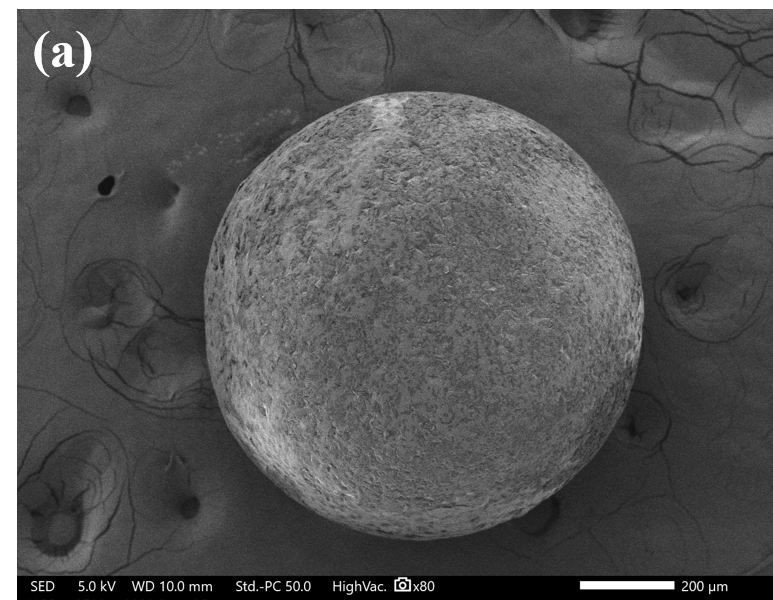


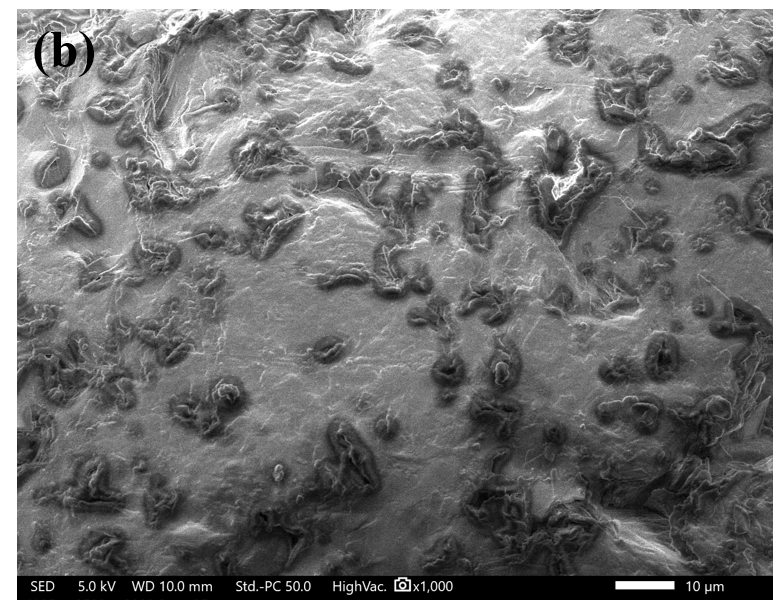


**Figure S6.** FE-SEM images of PE microspheres in cleansing cream B after HNO_3_ digestion. (a) At low magnification. (b) At high magnification.


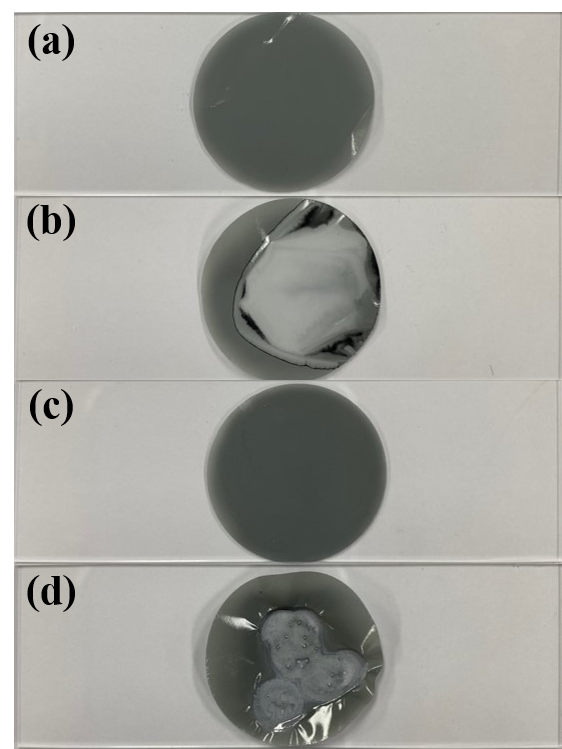


**Figure S7.** The black PC filter images. (a) With any solvent applied. (b) With a few drops of methanol. (c) With a few drops of *n*-hexane. (d) With a few drops of chloroform.


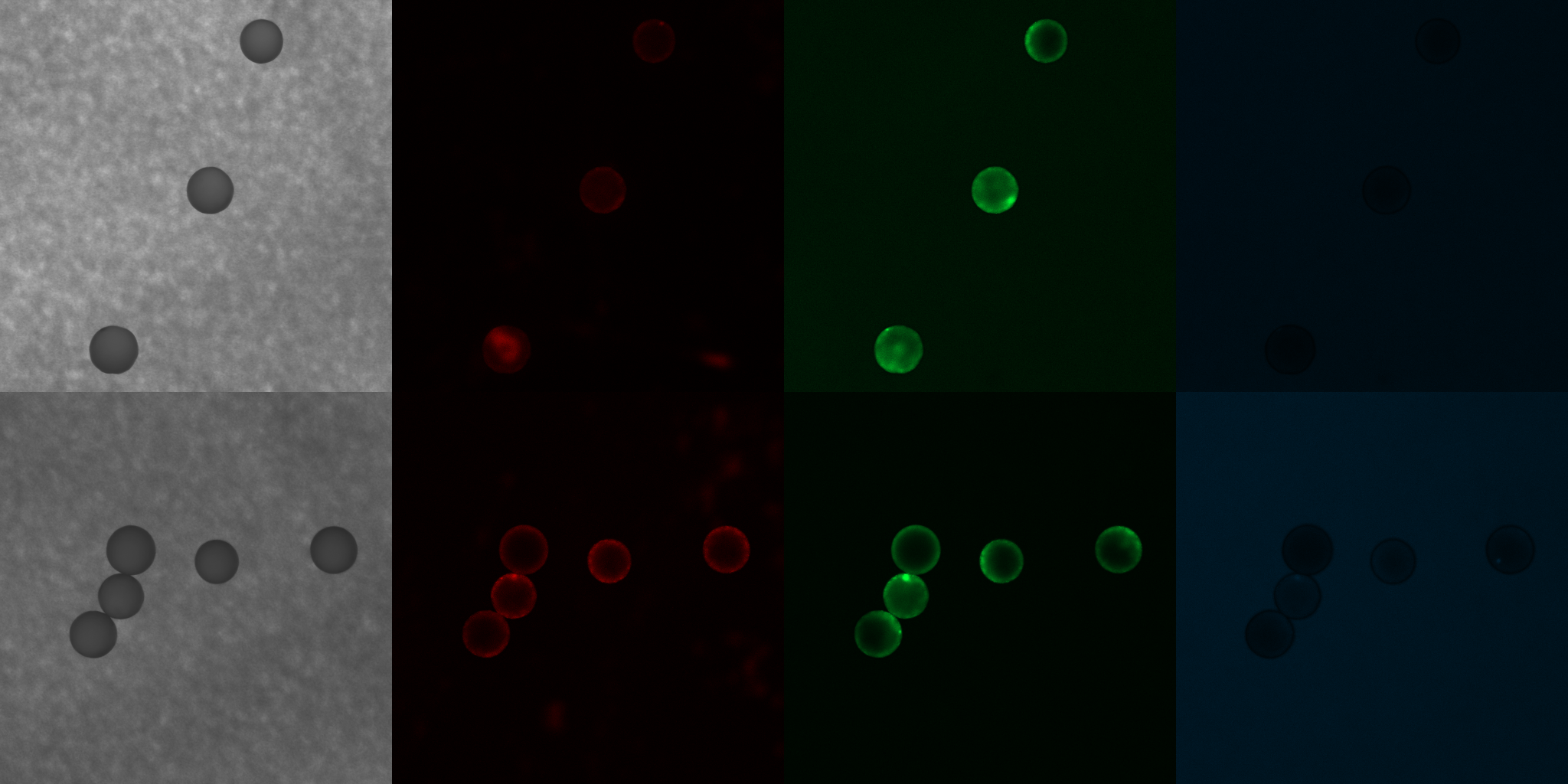


**Figure S8.** Fluorescent images with shadow, at red wavelengths, green wavelengths and blue wavelengths.


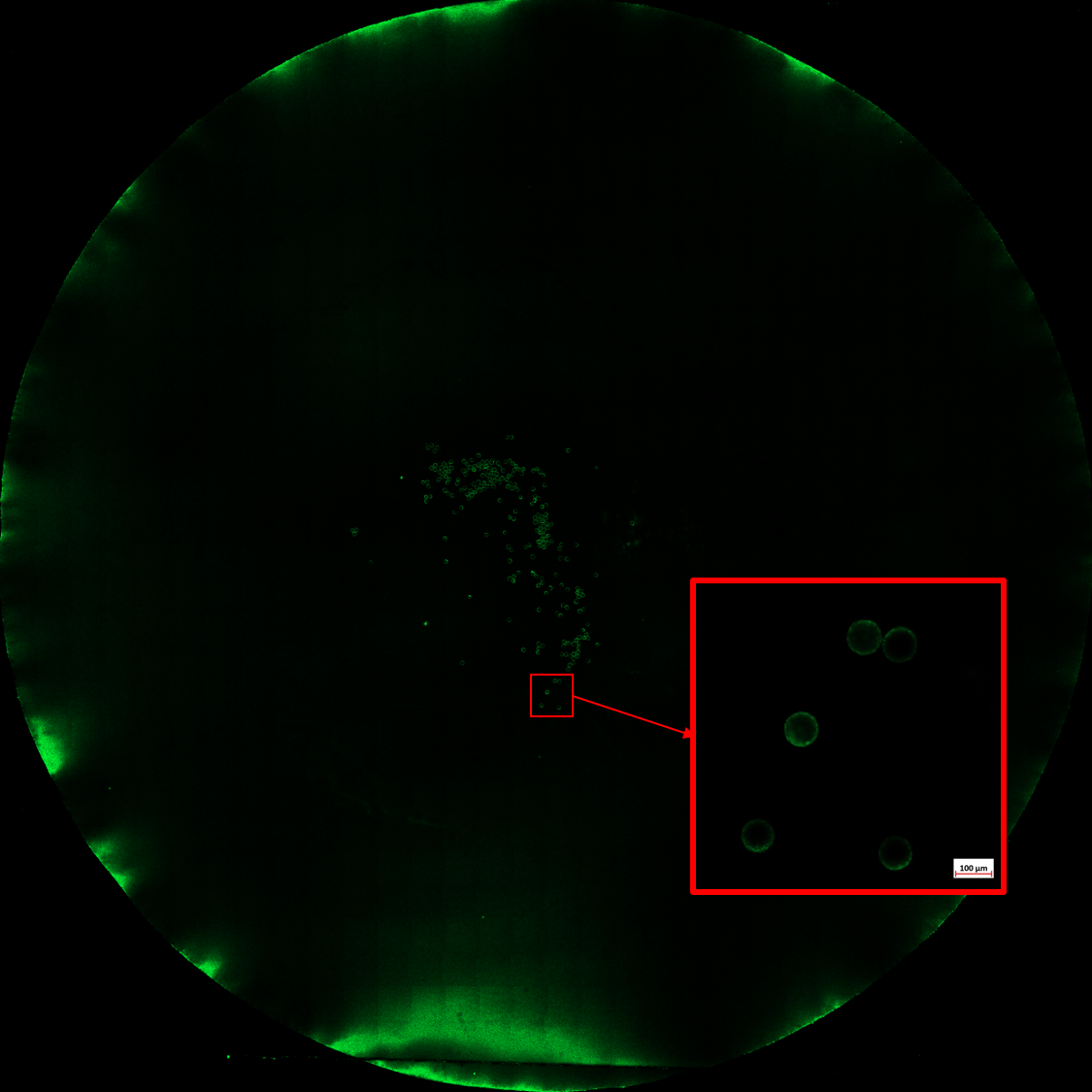


**Figure S9.** Overall fluorescent image of black PC filter.

**Table S1.** Prohibited microplastic ingredients in cosmetic products in Korea.

| No. | Name | Formular | No. | Name | Formular |
| --- | --- | --- | --- | --- | --- |
| 1 | Polyethylene (PE) | 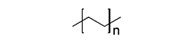 | 9 | Poly(tetrafluoroethylene) (PTFE) | 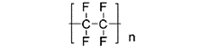 |
| 2 | Polyamide 6 (Nylon-6) | 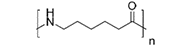 | 10 | Polyurethane (PU) | 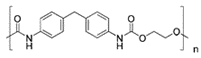 |
| 3 | Polyamide 12  (Nylon-12) | 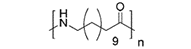 | 11 | Poly(sodium acrylate) (PA) | 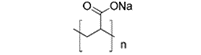 |
| 4 | Poly(butylene terephthalate) (PBT) | 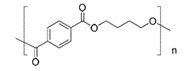 | 12 | Acrylates copolymer | 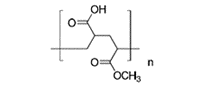 |
| 5 | Poly(methacrylic acid methyl ester) (PMMA) | 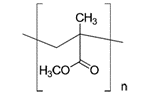 | 13 | Poly(ethylene-co-ethyl acrylate) | 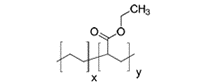 |
| 6 | Poly(ethylene terephthalate) (PET) | 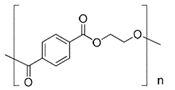 | 14 | Poly(ethylene-co-methyl acrylate) | 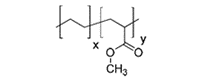 |
| 7 | Polypropylene (PP) | 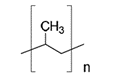 | 15 | Polystyrene-block-poly(ethylene-ran-butylene)-block-polystyrene | 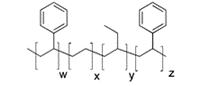 |
| 8 | Polystyrene (PS) | 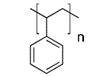 | 16 | Styrene acrylates copolymer | 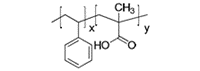 |

**Table S2.** Digestion efficiency and recovery rates of cleansing cream A-C through KOH digestion.

|  | Cleansing cream A | Cleansing cream B | Cleansing cream C |
| --- | --- | --- | --- |
| Digestion efficiency (%) | 98.32±1.06 | 98.65±1.14 | 97.64±1.05 |
| Recovery rates of MPs (%) | 95.00±10.49 | 95.50±5.22 | 96.50±6.34 |
